# Lymphatic trafficking of immune cells and insights for cancer metastasis

**DOI:** 10.1007/s10585-023-10229-3

**Published:** 2023-08-22

**Authors:** David G. Jackson

**Affiliations:** Radcliffe Department of Medicine, MRC Weatherall Institute of Molecular Medicine, University of Oxford, Oxford, OX3 9DS UK

**Keywords:** Lymphatic, Immune cell, Tumour metastasis, LYVE-1, Hyaluronan, Glycocalyx

## Abstract

Most cancers and in particular carcinomas metastasise via the lymphatics to draining lymph nodes from where they can potentially achieve systemic dissemination by invasion of high endothelial blood venules (HEVs) in the paracortex [1, 2]. Currently however, the mechanisms by which tumours invade and migrate within the lymphatics are incompletely understood, although it seems likely they exploit at least some of the normal physiological mechanisms used by immune cells to access lymphatic capillaries and traffic to draining lymph nodes in the course of immune surveillance, immune modulation and the resolution of inflammation [3, 4]. Typically these include directional guidance via chemotaxis, haptotaxis and durotaxis, adhesion to the vessel surface via receptors including integrins, and junctional re-modelling by MMPs (Matrix MetalloProteinases) and ADAMs (A Disintegrin And Metalloproteinases) [5–7]. This short review focusses on a newly emerging mechanism for lymphatic entry that involves the large polysaccharide hyaluronan (HA) and its key lymphatic and immune cell receptors respectively LYVE-1 (Lymphatic Vessel Endothelial receptor) and CD44, and outlines recent work which indicates this axis may also be used by some tumours to aid nodal metastasis.

## Article

The majority of immune cells that traffic from peripheral tissues to afferent lymphatics effect their entry within the first 200 μm of initial blind-ended capillaries at the distinctive overlapping junctions between the oakleaf shaped endothelial cells [5]. Here, and to a certain extent in adjoining lymphatic pre-collectors the interdigitating membrane flaps of such cells are “buttoned” at their sides by the adherens-junction protein VE-cadherin (Vascular Endothelial cadherin) and tight junction proteins including Claudin-5, ZO-1 (Zonula Occludens-1), ESAM (Endothelial Selective Adhesion Molecule) and JAM-A (Junctional Adhesion Molecule-A), while their tips are lined with PECAM-1 (Platelet Endothelial Cell Adhesion Molecule-1, CD31), and the lymphatic endothelial HA receptor LYVE-1 [5, 8–10] to form discrete junctional “portals” of approximately 0.5–1 μm through which individual immune cells can enter by pushing and squeezing [5, 11–14]. As predicted from its location in such portals, we recently demonstrated that LYVE-1 plays a key role in the entry of antigen presenting Dendritic cells (DCs) and macrophages to initial capillaries by engaging with its ligand HA, anchored in the surface glycocalyx of the incoming immune cells by the closely related leucocyte receptor CD44 [15–17]. This role of LYVE-1 has similarities to the one played by CD44 in mediating the exit of T cells from inflamed blood capillaries. There however, the HA glycocalyx is present on the (luminal) endothelium, where it serves to capture activated circulating T cells (which also express CD44 but lack an HA coat) by forming a CD44:HA:CD44 sandwich [18–20]. Synthesized primarily by HA synthase-2 (HAS2) in DCs and macrophages, the nascent HA chains associate with CD44 in intracellular vesicles, from which the bound complexes are then exported to the immune cell surface to form a glycocalyx of some 200-300 nm thickness as observed by high resolution (Airyscan) confocal microscopy [17]. Conclusive evidence that LYVE-1:HA interactions can be critical for vessel entry in vivo came from studies tracking the migration of fluorescently labelled DCs in oxazolone hypersensitized skin, which demonstrated that *Lyve1* gene deletion or mAb blockade led to a peri-lymphatic accumulation of DCs in the dermis and a consequent delay or inhibition of their trafficking to downstream axial LNs respectively [16]. Likewise, deletion of CD44 or enzymatic depletion of the HA glycocalyx resulted in a marked reduction in DC nodal migration compared to controls. Moreover, the significance of LYVE-1 • HA mediated DC migration for in vivo immune function was underscored by experiments with mice immunised intradermally with influenza virus nucleoprotein or ovalbumin peptide as model vaccines. These showed that both *Lyve1* gene deletion and mAb blockade disrupted the generation of DC primed peptide-specific CD4 and CD8 T-cell responses in downstream lymph nodes, confirming that in vivo, the LYVE-1• HA mediated entry of immune cells to lymphatics can be rate-limiting for protective immunity ([16] and reviewed in Johnson et al. [14]). The critical importance of LYVE-1 for immune cell trafficking has been further reinforced by the discovery that the process is controlled by the Circadian clock gene *BMAL1* which directly regulates the expression of LYVE-1, CCL21 and CCR7 in lymphatic endothelium to facilitate maximal DC migration during the sleeping hours, when the immune activity of lymph nodes is at its highest [21, 22].

As regards the precise molecular mechanism of HA-mediated entry, we have observed that contact with the DC glycocalyx triggers redistribution of LYVE-1 into ring-like assemblages termed transmigratory cups in the underlying endothelium, owing to their similarity to structures that form around T-cells during exit across blood vascular endothelium [23–25]. Coincident with engaging LYVE-1 in these cups, we reported that migrating DCs undergo polarisation to form a uropod at the trailing edge, in which CD44 is concentrated with its bound HA glycocalyx, and a leading edge or lamellipodium that extends to explore the surrounding endothelial surface for potential sites of transit, most likely at tri-cellular junctions [17]. Hence, the adhesive interface between HA and LYVE-1 likely provides a flexible foothold for the DC to attach to the outer surface of a lymphatic capillary while the actomyosin machinery provides the tractive force for transit to the vessel lumen either through amoeboid motility or adhesion to integrins at the leading edge, directionally guided by a haptotactic gradient of the chemokine CCL21 in the perilymphatic matrix via its signaling receptor CCR7 [26]. Additionally, DCs can trigger localised discharge of CCL21 from lymphatic endothelium to guide their own transmigration, through Ca^2+^ triggered exocytosis of the chemokine from pre-stored depots in *trans* Golgi vesicles [27, 28]. Indeed, the LYVE-1 HA axis appears to regulate such discharge, insofar as engagement of DCs via LYVE-1 can induce CCL21 secretion from LECs, and release of CCL21 from dermal lymphatic capillaries is disrupted in *Lyve1*^*−/−*^ mice. (Johnson and Jackson unpublished). Besides DCs, our ongoing investigations have confirmed that tissue macrophages also utilize their HA glycocalyx to mediate exit from inflamed tissue to afferent lymphatics via LYVE-1, notably during the phase of resolution that enables restoration of normal homeostasis in tissues such as the infarcted heart and peritoneum [29, 30].

Of course, the LYVE-1 HA axis is not the only one to play a role in lymphatic entry. With the exception of DCs and macrophages, most other immune cell populations appear to lack a HA glycocalyx, and must instead initiate lymphatic transit by other mechanisms. In the case of T_EM_ and T_REG_ cells for example, it has been reported that entry involves the chemotactic lipid Sphingosine 1-P along with lymphotoxin (LTα1β3) and its receptor LTβR [31, 32]. Neutrophils, the first responders to tissue injury can adhere to the vessel endothelium *via* β2 integrins and transmigrate through the combined actions of the matrix metalloproteinases MMP8 and MMP9, the serine protease neutrophil elastase and the arachidonate-derived chemorepellent, 12,hydroxyeicosatetraenoate (12(S)HETE) that together promote junctional retraction [33, 34]. Furthermore, it appears likely that both these populations, which migrate primarily in response to inflammation, can enter dermal lymphatics through downstream collectors which lack LYVE-1 and have conventional tight (zippered) endothelial junctions rather than buttoned junctions. Here, it has been reported that the process of entry is mediated by adhesion between integrins on the immune cells and VCAM-1 (Vascular Endothelial Adhesion Molecule – 1) and ICAM-1 (Intercellular Adhesion Molecule – 1) in the inflamed endothelium [35–40]. Notably, it was shown that such integrin dependent transit through downstream collectors is also used by DCs, most likely to accelerate their passage to downstream lymph nodes in inflamed tissues [35]. Indeed integrin-mediated adhesion may even combine with HA-mediated adhesion for DC entry at initial capillaries or pre-collectors, as both ICAM-1 and VCAM-1 have been observed to co-localise with LYVE-1 during transmigration via transmigratory cups [36, 37]. Hence, while the individual mechanisms used to initiate lymphatic entry can differ between immune cell types, they can also vary according to the tissue context. Moreover, the overall process actually involves a series of steps that likely involve contributions from a number of different adhesion receptors and their ligands including ALCAM (Activated Leukocyte Cell Adhesion Molecule), L1CAM, CD99, Mannose receptor, 41-BB/CD137 and CLEVER-1(Combined Lymphatic Endothelial and Vascular Endothelial Receptor − 1), as well as chemokines, chemoattractants and proteinases that together activate signaling pathways leading to the loss of VE-cadherin and junctional retraction [7, 41].

The use of the LYVE-1·HA adhesion axis by DCs and macrophages for lymphatic entry can be reconciled with the unusual mechanics of the binding interaction and its tissue context. Unlike CD44, to which individual HA chains bind through conventional ”sticking” interactions, the HA chains can “slide” through the cleft-like binding site in LYVE-1, a process that likely equips DCs and macrophages for their fluid-like crawling on lymphatic endothelium and subsequent transmigration in the low shear environment of initial lymphatics [10, 42]. Likewise, the absence of an HA glycocalyx from T cells and neutrophils likely reflects the fact they reside mainly in the blood circulation where the firmer binding properties of CD44 enable their extravasation at sites of tissue injury by adhesion to endothelial HA in the face of rapid blood flow.

## The HA pericellular coat in tumours - a potential role in nodal metastasis?

Besides being a feature of tissue based immune cell populations as already discussed, the assembly of an HA glycocalyx or pericellular coat is also a property of other less motile cell types such as fibroblasts, chondrocytes and epithelial cells, where the large polyanionic nature of the sugar polymer acts as a barrier modulating cell:cell contact, as well as a means of tissue anchorage via proteoglycans in the extracellular matrix [43]. Importantly, the HA pericellular coat can be radically altered during cell transformation. Notably, its expansion through upregulation of HAS gene expression and HA deposition are hallmarks of epithelial to mesenchymal transition [44], a fundamental process in carcinoma progression by which tumour cells acquire enhanced migratory capacity [45]. Furthermore, elevated levels of both HA and CD44 are common amongst “aggressive” and metastatic cancers and associated with poor survival [46, 47].

There is now growing evidence to suggest this tumour cell HA coat can contribute to metastasis by facilitating lymphatic invasion of either individual tumour cells or tumour emboli via LYVE-1, perhaps in a similar manner to the trafficking of immune cells. Most if not all tumours grow in close proximity to host lymphatics or generate new peritumoral or even intratumoral lymphatics, both of which have LYVE-1 lined endothelial junctions. Moreover, in immunostained paraffin tissue sections of breast cancer, a CD44-associated pericellular HA matrix has been observed on tumour emboli invading such lymphatic vessels [48], while in vitro cultured breast tumour lines have been reported to adhere via HA to LYVE-1 in transfected fibroblasts [49]. Extending upon these observations, our own more recent studies indicate that assembly of a dense CD44 anchored HA glycocalyx is a consistent feature of metastatic MDA MB 231 and MCF-7 breast carcinomas but not poorly metastasizing SKBR3 cells, and that MDA MB 231 and MCF-7 cells both adhere and transit human LEC monolayers via LYVE-1 transmigratory cups similar to those formed by transmigrating DCs (Johnson and Jackson unpublished). Curiously, such findings chime with those from a previous ultrastructural study in mice where detailed electron microscopic imaging suggested that prostate adenocarcinomas, colon carcinomas and B16 melanomas extend filopodia-like protrusions towards lymphatic vessels during invasion, and that the endothelial cells simultaneously extend transmigratory cup-like projections around the transiting tumour cells [50]. Moreover, in further studies with the mouse 4T1 mammary carcinoma line that can spread directly from the first draining lymph nodes to the blood circulation in Balb/c animals by invading HEVs [51, 52] we found the initial invasion of local lymphatics and metastasis to draining inguinal lymph nodes is both reliant on HA and LYVE-1 and severely impaired in *Lyve1*^*−/−*^*mice* (Johnson LA and Jackson DG unpublished). It is tempting to speculate that subsequent colonisation of these inguinal nodes by 4T1 as well as invasion of blood vessels for systemic dissemination of the tumour involves yet further interactions with LYVE-1, given its abundance in the sinuses that must be traversed to reach the HEVs; however, this remains to be investigated.

**Fig. 1:**
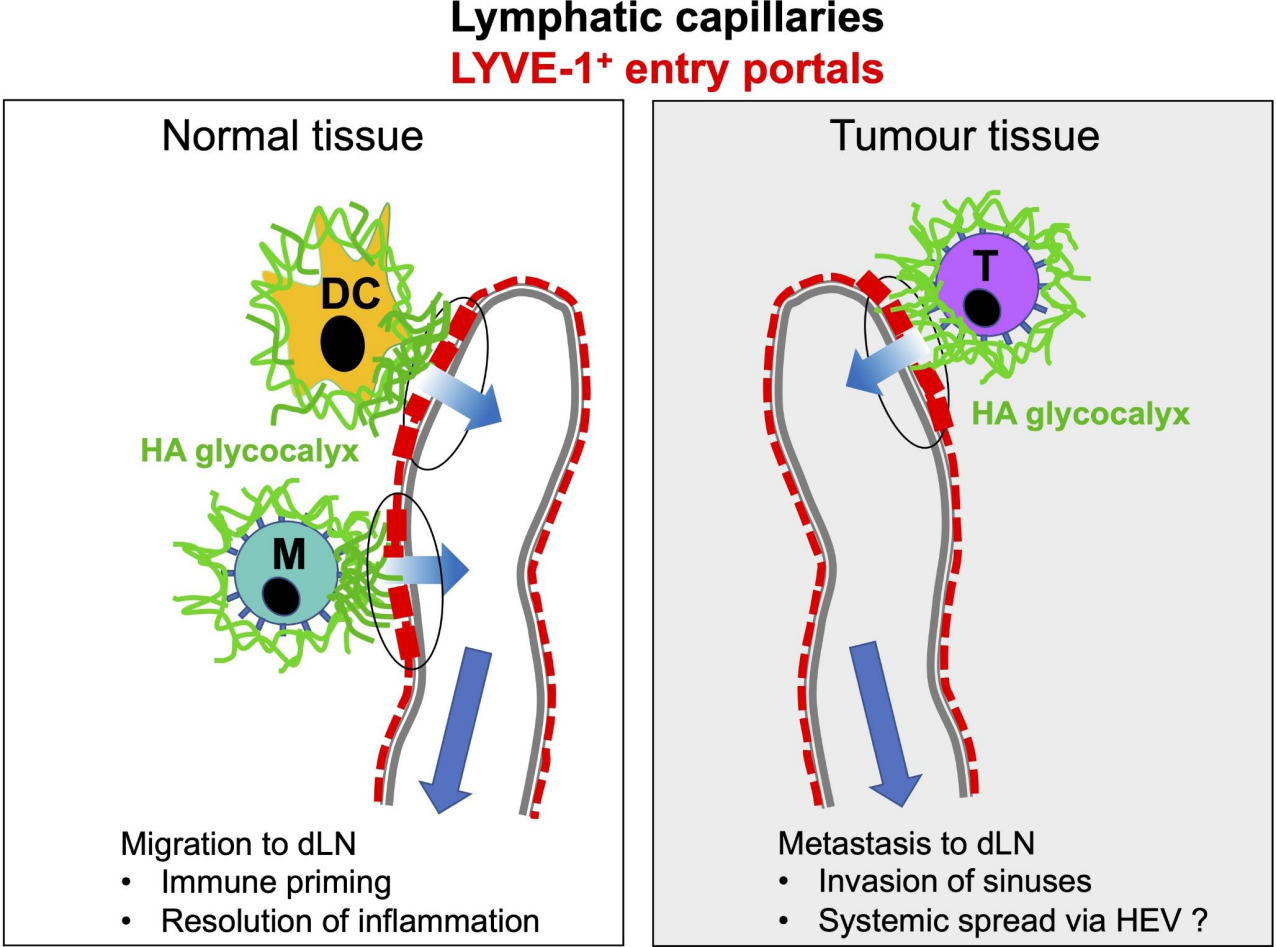
**Involvement of LYVE-1 and hyaluronan glycocalyx in entry of immune cells and tumours to initial lymphatics**. Pictorial images showing potential similarity between mechanisms used by normal immune cells and tumour cells to enter lymphatic capillaries by means of LYVE-1 and hyaluronan glycocalyx. Panels depict a blind-ended lymphatic in each case containing multiple LYVE-1 lined entry portals (red stippling). Entry of dendritic cells (DC), macrophages (M) and tumour cells (T) involves clustering of the glycocalyx and formation of LYVE-1 enriched transmigratory cups that facilitate transit to the vessel lumen

In summary, both tissue-based immune cells and metastatic tumours employ similar adhesive interactions between their endogenous HA surface glycocalyx and the endothelial receptor LYVE-1 to enter afferent lymphatics and migrate to downstream lymph nodes - the former to promote immunity and its resolution and the latter for systemic dissemination (Fig. 1). Accordingly, LYVE-1 is a potential target for the therapeutic blockade of both immune based disorders and cancer metastasis. Regarding the latter however, one important caveat is that lymphatic trafficking is pivotal to the generation of anti-tumour immunity and its disruption would therefore be undesirable. Hence, LYVE-1 blockade might be most effective for the treatment of early-stage cancers, when administered after primary tumour resection, prior to nodal dissemination and local immune activation. Further studies will no doubt help to clarify these issues.

## Data Availability

Data sharing not applicable to this article as no datasets were generated or analysed during the current study.
